# Learning more by sampling less: subsampling effects are model specific

**DOI:** 10.1186/1471-2202-14-S1-P414

**Published:** 2013-07-08

**Authors:** Viola Priesemann, Michael Wibral, Jochen Triesch

**Affiliations:** 1 Max Planck Institute for Brain Research, Frankfurt, 60486, Germany; 2 Frankfurt Institute for Advanced Studies, Frankfurt, 60438, Germany; 3 Goethe University, MEG Unit, Frankfurt, 60325, Germany

## 

When studying real world complex networks, one rarely has full access to all their components. As an example, the central nervous system of the human consists of 10^11 ^neurons which are each connected to thousands of other neurons [1]. Of these 100 billion neurons, at most a few hundred can be recorded in parallel. Thus observations are hampered by immense subsampling. While subsampling does not affect the observables of single neuron activity, it can heavily distort observables which characterize interactions between pairs or groups of neurons [2]. Without a precise understanding how subsampling affects these observables, inference on neural network dynamics from subsampled neural data remains limited.

We systematically studied subsampling effects in three self-organized critical (SOC) models, since this class of models can reproduce the spatio-temporal activity of spontaneous activity observed *in vivo *[2,3]. The models differed in their topology and in their precise interaction rules. The first model consisted of locally connected integrate- and fire units, thereby resembling cortical activity propagation mechanisms [2]. The second model had the same interaction rules but random connectivity [4]. The third model had local connectivity but different activity propagation rules [5]. As a measure of network dynamics, we characterized the spatio-temporal waves of activity, called avalanches. Avalanches are characteristic for SOC models and neural tissue [6]. Avalanche measures *A *(e.g. size, duration, shape) were calculated for the fully sampled and the subsampled models. To mimic subsampling in the models, we considered the activity of a subset of units only, discarding the activity of all the other units.

Under subsampling the avalanche measures *A *depended on three main factors: First, *A *depended on the interaction rules of the model and its topology, thus each model showed its own characteristic subsampling effects on *A*. Second, *A *depended on the number of sampled sites *n*. With small and intermediate *n*, the true *A¬ *could not be recovered in any of the models. Third, *A *depended on the distance *d *between sampled sites. With small *d, A *was overestimated, while with large *d, A *was underestimated.

Since under subsampling, the observables depended on the model's topology and interaction mechanisms, we propose that systematic subsampling can be exploited to compare models with neural data: When changing the number and the distance between electrodes in neural tissue and sampled units in a model analogously, the observables in a *correct *model should behave the same as in the neural tissue. Thereby, incorrect models can easily be discarded. Thus, systematic subsampling offers a promising and unique approach to model selection, even if brain activity was far from being fully sampled.
